# Artifactual Hypoglycaemia in Systemic Sclerosis and Raynaud's Phenomenon: A Clinical Case Report and Short Review

**DOI:** 10.1155/2016/7390927

**Published:** 2016-12-28

**Authors:** RH Bishay, A. Suryawanshi

**Affiliations:** ^1^Department of Endocrinology and Metabolism, Concord Repatriation General Hospital, Concord, Sydney, NSW 2139, Australia; ^2^Sydney Medical School, University of Sydney, Sydney, NSW 2005, Australia

## Abstract

*Background*. Artifactual hypoglycaemia, defined as a discrepancy between glucometer (capillary) and plasma glucose levels, may lead to overtreatment and costly investigations. It is not infrequently observed in patients with Raynaud's phenomenon due to vascular capillary distortion, yet this is clinically underappreciated.* Case Report*. We report a 76-year-old woman with systemic sclerosis and Raynaud's phenomenon, who presented with upper gastrointestinal bleeding and found to have concomitant persistent hypoglycaemia (1.0–2.7mmol/L) on a point-of-care glucometer in the absence hypoglycaemic symptoms. She underwent a 2-week hospital admission, repeated glucose monitoring, hydrocortisone replacement and dextrose infusions, with consequent hyperglycaemia on plasma measurements. Clinically, she did not satisfy* Whipple's* triad and radiological investigations failed to identify pituitary or pancreatic pathology. A 72-hour fast was negative for hyperinsulinaemia or exogenous insulin use and her sulphonylurea metabolite urinary screen was negative.* Discussion*. Treatment of low capillary blood glucose is usually met with clinical impetus to treat, even when hypoglycaemic symptoms are lacking. The correct diagnosis may have been achieved had there been an observation of her cold hands, scleroderma facies, and consideration of the likely distorted peripheral microvasculature. Early identification of this presumably rare clinical scenario may have prevented overtreatment, altered methods of monitoring, and avoided unnecessary investigations.

## 1. Background

Systemic sclerosis (previously known as scleroderma) is a multisystem disease with complex pathophysiology that results from extensive fibrosis, abnormal vascular tone, and autoimmunity to multiple cellular epitopes [1]. Raynaud's phenomenon, where there is an exaggerated vasoconstriction of peripheral blood vessels, can precede the diagnosis for many years [1]. With few exceptions, there are a limited number of reports in the literature of artifactual hypoglycaemia, also used interchangeably (albeit erroneously) with pseudohypoglycaemia, in patients with Raynaud's phenomenon and/or systemic sclerosis [2, 3] due to the abnormal blood transit time in peripheral capillaries. The following case illustrates the need for high clinical suspicion in patients with whom critically low glucose levels are obtained by capillary blood glucose measurements yet deny the classical symptoms of hypoglycaemia.

## 2. Case Report

A 76-year-old Caucasian female presented to the emergency department from home with a 1-day history of symptomatic anaemia and fatigue in the context of melaena. She denied respiratory or cardiac symptoms or a history of weight loss, painful defecation, obstruction, use of nonsteroidal anti-inflammatory medications, a history of upper gastrointestinal ulceration or* H. pylori* infection. Her past medical history was significant for centromere antibody positive systemic sclerosis with features of the CREST syndrome, inclusive of pulmonary fibrosis, severe pulmonary hypertension, Raynaud's phenomenon, gastric antral vascular ectasia, gastritis, and reflux oesophagitis; she also had postmenopausal osteoporosis exacerbated by corticosteroid use. Her medications included long-term prednisone 15 mg daily, bosentan 62.5 mg BD, furosemide 20 mg daily, esomeprazole 20 mg daily, denosumab 60 mg S/C six-monthly, cholecalciferol 1000 U daily, and calcium carbonate 600 mg daily. She lived alone, is a life-long nonsmoker, and consumes 1-2 standard drinks of alcohol daily. She is independent of daily activities.

On examination, she appeared unwell and thin (body mass index 17.5 kg/m^2^) with cool peripheries and scleroderma facies. Blood pressure was 70 mmHg systolic, heart rate was 70 beats per minute, and cardiorespiratory examination revealed fine, bibasal crackles consistent with pulmonary fibrosis. Gastrointestinal examination noted melaena on* per rectal* examination but no stigmata of chronic liver disease.

Initial point-of-care capillary glucometer readings revealed glucose levels of 1.7 mmol/L, 1.3 mmol/L, and then 1.0 mmol/L taken at 15-minute intervals. The patient denied sympathetic (i.e., palpitations, diaphoresis, and tremor) or neuroglycopenic (i.e., confusion, headache, visual changes, and nausea) symptoms associated with hypoglycaemia and denied a history of diabetes, exogenous use of insulin, or insulin secretagogues. Investigations revealed a formal glucose of 28.3 mmol/L. She also had normocytic anaemia (haemoglobin of 63 g/L [120–150]) with preserved renal function. Platelet count was normal with mildly deranged gamma-glutamyl transferase (130 U/L, [<35]), international normalised ratio (INR) (1.3), and reduced albumin (27 g/L [38–48]). Haemolytic anaemia screen was negative and iron studies revealed iron deficiency (transferrin saturation 13%  [15–50], ferritin 41 ug/L [20–300]). C-reactive protein was 16.3. Thyroid function and lipase were within normal limits.

Acute management included two stat boluses of dextrose, with capillary blood glucose levels temporarily increasing from 1.0 mmol/L to 7.0 mmol/L and remaining unexpectedly low-normal (4.7 to 5.5 mmol/L). The patient was admitted to the high-dependency unit and, given her history of corticosteroid use and presumptive diagnosis of adrenal insufficiency, was given stress hydrocortisone doses, moderate fluid resuscitation, intravenous proton-pump inhibitor infusion, and several units of transfused packed red blood cells. Her chest X-ray revealed longstanding ill-defined ground glass opacities. The patient underwent a gastroscopy which diagnosed severe ulcerative esophagitis as the cause of her bleeding. She had a 2-week admission with several medical review alerts for asymptomatic hypoglycaemia. Capillary glucose levels were consistently between 1.7 and 3.8 mmol/L. The patient eventually recovered well and was discharged on esomeprazole 20 mg BD without further melaena.

**Figure 1:**
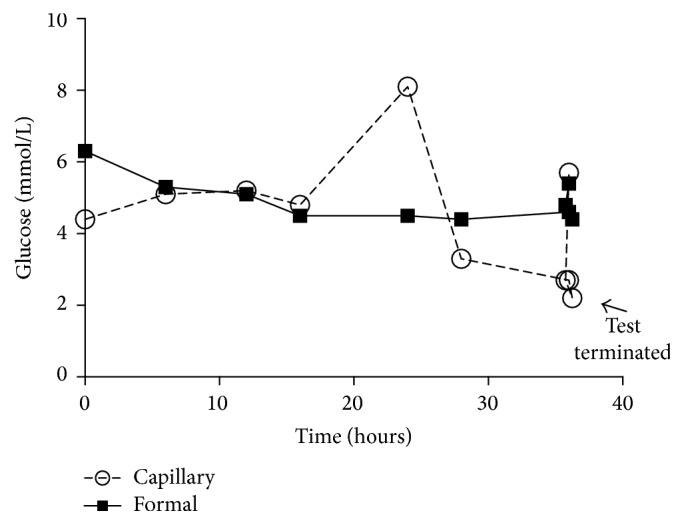
A seventy-two-hour fast protocol revealed a discrepancy between capillary (point-of-care glucometer) and venous blood glucose levels. The test was terminated early due to low capillary glucose levels (2.2 mmol/L).

The patient underwent a 72-hour fast protocol that was terminated early due to hypoglycaemia (≤2.2 mmol/L) on capillary glucose monitoring (Figure 1). Her pituitary profile (adrenocorticotrophic hormone, morning cortisol, prolactin, growth hormone, insulin growth factor-1, and thyroid function) was within normal limits (not shown). Gonadotrophins were consistent with menopause. Given her anaemia, a computed tomographic imaging of her abdomen was performed to assess for malignancy, which was normal. Magnetic resonance imaging of her brain did not reveal pituitary apoplexy or other compressive sellar or suprasellar lesions.

## 3. Discussion

Artifactual hypoglycaemia has no clear definition but has been proposed to be defined as a discrepancy between capillary and plasma blood glucose levels, due to either differing laboratory techniques or other patient factors, as in our index patient. Pseudohypoglycaemia in contrast is defined as the presence of typical sympathetic or neuroglycopenic symptoms in the presence of a blood glucose level >3.9 mmol/L [4]. Artifactual hypoglycaemia in patients with systemic sclerosis is thought to be due to low capillary blood glucose levels due to reduced capillary flow, leading to deceleration of glucose transit and subsequent increased uptake of glucose by local tissues [5]. Patients with systemic sclerosis have vascular injury affecting primarily small vessels and arterioles that occurs early in the disease process, which can distort the integrity of the endothelial lining and results in progressive thinning of capillaries and loss of blood vessels [6, 7].

Confounding aspects contributing to her low capillary blood glucose levels included blood loss due to melaena and hypotension, which may have caused peripheral shunting of blood volume, as well as her long history of glucocorticoid use and possible adrenal insufficiency. However, even after correcting both of these conditions, the capillary hypoglycaemia persisted. In fact, relatively large volumes of dextrose and intravenous hydrocortisone may have precipitated worsening hyperglycaemia and the possible development of hyperosmolar hyperglycaemic state or diabetic ketoacidosis. Thus, the correct diagnosis is paramount to avoid further inpatient metabolic disturbance.

Previous case reports of patients with artifactual hypoglycaemia in the context of systemic sclerosis with Raynaud's phenomenon all seemingly had very low fasting and random capillary, point-of-care measurements using a glucometer, ranging between 0.61 mmol/L and 3 mmol/L [2, 3, 8, 9]. In all cases, blood samples taken from either the forearm or a vein from the cubital fossa were normal however. The majority of patients underwent screening for hypopituitarism with a tetracosactide test as well as 72-hour fast to exclude endogenous hyperinsulinaemia, as in our index patient. Obtaining symptoms from history is critical as most patients will deny the classic sympathetic and neuroglycopenic symptoms of hypoglycaemia and therefore do not satisfy* Whipple's* triad.* Whipple's* triad confirms true hypoglycaemia secondary to endogenous hyperinsulinaemia and includes the following criteria: (i) typical symptoms of hypoglycaemia with (ii) a low plasma glucose measured at the time of the symptoms and (iii) relief of these symptoms when the glucose is raised to normal; none of these criteria were met by the patient.* Whipple's* triad is also a necessary to establish grounds for a 72-hour fast to exclude the presence of an insulinoma, the commonest functioning neuroendocrine tumour arising from the pancreas. In some cases, however, symptoms alone cannot be relied upon as many patients may falsely attribute nonspecific complaints to hypoglycaemia [8]. Other investigations including insulin, proinsulin, glucagon, cortisol, growth hormone, and hydroxybutyrate (a surrogate marker of starvation and inversely related to insulin secretion) are expectedly normal. All patients however should be assessed for surreptitious insulin or sulphonylurea use, the latter with a urinary sulphonylurea metabolite analysis.

Although the terms artifactual hypoglycaemia (coined in the 1960s) and pseudohypoglycaemia continue to be used interchangeably, there is a potential for some confusion since the American Diabetes Association and the Endocrine Society workgroup favoured the use of the term pseudohypoglycaemia in 2013 [4]. Its definition is problematic in at least three scenarios: (1) it does not differentiate between pseudohypoglycaemia from patients with symptoms attributable to other causes and falsely low glucose readings from a point-of-care glucometer, as in our index patient; (2) patients who are chronically hyperglycaemic who have sudden correction of their glucose to normal levels may have similar symptoms in the presence of normal capillary glucose levels; and (3) patients with poor glycaemic control may, in the context of a condition or a medication that affects capillary glucose readings, be falsely reassured of optimal glycaemic control. Therefore, the authors support the distinction between pseudohypoglycaemia and artifactual hypoglycaemia, the latter being better suited to our case.

It is notable to mention other causes of pseudohypoglycaemia, which are summarised in Table 1, such as disorders that lead to impaired capillary blood flow, increased glycolysis, hyperviscosity and medications [10] as well as preanalytic factors. Leukemia, in particular, can lead to artifactual hypoglycaemia due to the increased glucose metabolism by a large number of leukocytes.

In summary, clinicians should be mindful of the limitations of point-of-care blood glucose measurements (i.e., glucometer). In general, low capillary glucose levels (e.g., <3 mmol/L) without associated sympathetic or neuroglycopenic symptoms should prompt formal blood glucose testing concomitant with a capillary glucose level to assess for a difference, as well as a reasonable search for other possible causes. Artifactual hypoglycaemia is inadvertently common in patients with systemic sclerosis due to abnormal capillary blood flow; hence heightened vigilance is required in interpreting capillary glucose values. Currently, there is no consensus on establishing a distinction between pseudohypoglycaemia and artifactual hypoglycaemia, though confirmation of the latter can help alleviate the unnecessary burden of multiple investigations, taxing healthcare expenditure, and provocation of patient anxiety that often accompanies these seemingly well patients.

## Figures and Tables

**Table 1:** Causes of pseudohypoglycaemia or artifactual hypoglycaemia.

Mechanism	Examples
Impaired capillary blood flow	Raynaud's phenomenon, acrocyanosis, peripheral vascular disease, Eisenmenger syndrome, circulatory shock, hypothermia
Increased glycolysis	Leukemia, polycythemia vera
Hyperviscosity	Plasma cell dyscrasias, for example, multiple myeloma, monoclonal gammopathy of unknown significance, Waldenstrom macroglobulinaemia
Medication	Ascorbic acid, dopamine, mannitol, acetaminophen
Preanalytic factors	Delay in separation of plasma from formed blood contents

## Additional Points

*Novelty Statement*. (i) Artifactual hypoglycaemia has real clinical implications, that is, when point-of-care glucometer readings are incorrect or misleading. (ii) Patients with Raynaud's phenomenon may display persistent artifactual hypoglycaemia due to distorted peripheral vasculature; this observation however is underrecognised in clinical practise and scarcely reported in literature. (iii) Costly monitoring, aggressive management, and radiological examinations often accompany the finding of persistent capillary hypoglycaemia in patients with Raynaud's phenomenon; this should be replaced with high clinical suspicion and consideration of a secondary cause of hypoglycaemia. (iv) In general, secondary causes of hypoglycaemia should be considered if the hypoglycaemia is persistent, symptoms are lacking, or conventional treatment is ineffective.
